# Characterization of Biofilm Formation in *[Pasteurella] pneumotropica* and *[Actinobacillus] muris* Isolates of Mouse Origin

**DOI:** 10.1371/journal.pone.0138778

**Published:** 2015-10-02

**Authors:** Martin Sager, W. Peter M. Benten, Eva Engelhardt, Christina Gougoula, Laurentiu Benga

**Affiliations:** Central Animal Research Facility, Heinrich—Heine—University, University Hospital, Düsseldorf, Germany; Indian Institute of Science, INDIA

## Abstract

*[Pasteurella] pneumotropica* biotypes Jawetz and Heyl and *[Actinobacillus] muris* are the most prevalent *Pasteurellaceae* species isolated from laboratory mouse. However, mechanisms contributing to their high prevalence such as the ability to form biofilms have not been studied yet. In the present investigation we analyze if these bacterial species can produce biofilms *in vitro* and investigate whether proteins, extracellular DNA and polysaccharides are involved in the biofilm formation and structure by inhibition and dispersal assays using proteinase K, DNase I and sodium periodate. Finally, the capacity of the biofilms to confer resistance to antibiotics is examined. We demonstrate that both *[P*.*] pneumotropica* biotypes but not *[A*.*] muris* are able to form robust biofilms *in vitro*, a phenotype which is widely spread among the field isolates. The biofilm inhibition and dispersal assays by proteinase and DNase lead to a strong inhibition in biofilm formation when added at the initiation of the biofilm formation and dispersed pre-formed *[P*.*] pneumotropica* biofilms, revealing thus that proteins and extracellular DNA are essential in biofilm formation and structure. Sodium periodate inhibited the bacterial growth when added at the beginning of the biofilm formation assay, making difficult the assessment of the role of β-1,6-linked polysaccharides in the biofilm formation, and had a biofilm stimulating effect when added on pre-established mature biofilms of *[P*.*] pneumotropica* biotype Heyl and a majority of *[P*.*] pneumotropica* biotype Jawetz strains, suggesting that the presence of β-1,6-linked polysaccharides on the bacterial surface might attenuate the biofilm production. Conversely, no effect or a decrease in the biofilm quantity was observed by biofilm dispersal using sodium periodate on further biotype Jawetz isolates, suggesting that polysaccharides might be incorporated in the biofilm structure. We additionally show that *[P*.*] pneumotropica* cells enclosed in biofilms were less sensitive to treatment with amoxicillin and enrofloxacin than planktonic bacteria. Taken together, these findings provide a first step in understanding of the biofilm mechanisms in *[P*.*] pneumotropica*, which might contribute to elucidation of colonization and pathogenesis mechanisms for these obligate inhabitants of the mouse mucosa.

## Introduction

Bacterial biofilms are structured aggregations of bacterial cells, encased in a self synthesized extracellular matrix that may consist of proteins, nucleic acids and polysaccharides [1]. Biofilm production occurs in a multi-step process in which the bacteria adhere to a surface and then produce the extracellular matrix which confer them a firmer adherence [2]. The biofilms are recognized as complicating factors in many bacterial infections and one of the reasons why treatment with antibiotics fails [3]. Moreover, the biofilms enhance the resistance of bacteria to host immune defense mechanisms [4].

*[Pasteurella] pneumotropica* biotypes Jawetz and Heyl and *[Actinobacillus] muris* are the most prevalent *Pasteurellaceae* species isolated from laboratory mouse [5]. Although described several decades ago, these species are expected to undergo taxonomic changes soon, since they have not been formally classified under genera due to insufficient knowledge regarding their taxonomy [6]. *[P*.*] pneumotropica*, the most well-known member of the rodent *Pasteurellaceae*, has two biovars, Jawetz and Heyl, which show a few phenotypic differences [7, 8], but are regarded as two distinct species based on the 16S rDNA and *gyrB* sequences [9–12].

*[P*.*] pneumotropica* seems to possess the most pathogenic potential among the rodent *Pasteurellaceae* and is associated with several pathogenic aspects in laboratory rodents such as inflammation and abscess formation in a majority of tissues [13–16]. In addition, *[P*.*] pneumotropica* can occasionally cause disease in immunodeficient [17], as well as in immunocompetent humans [18]. Moreover, subclinical infection with *[P*.*] pneumotropica* may trigger immune mechanisms with possible interference on research using infected mice [19]. No pathogenic potential could be attributed to date to *[A*.*] muris* [20, 21].

Despite the biological and economic importance of *[P*.*] pneumotropica* in relation to experimental animals, the virulence factors and the pathogenesis of *[P*.*] pneumotropica* infections are largely unknown. Nevertheless, in the last years these infectious agents received an increased importance and putative virulence factors such as hemolysin-like proteins similar to RTX toxin [22] and their immunogenic role have been considered [23]. The immunogenic and protective role of several outer membrane proteins of *[P*.*] pneumotropica* have also been studied recently [24]. The increasing interest in *[P*.*] pneumotropica* characterization is also underlined by the report of the first genome sequence for this species [25]. Although *[P*.*] pneumotropica* as a pathogen was less investigated, its potential as a model organism in the mouse, to study some pathogenic processes such as periodontal bone resorption [26, 27] or the role of toll-like receptor 4 in the gram-negative bacterial pneumonia [28] has been recognized. The ability of *[P*.*] pneumotropica* to evade the human complement was also documented recently [29]. It is known that many pathogenic *Pasteurellaceae* species of big animals are strictly adapted to their host species. For this reason, we believe that the mouse is not an ideal animal model to study pathogenesis aspects like colonization for big animal *Pasteurellaceae* and that *[P*.*] pneumotropica* can serve as prototype for investigations of other related *Pasteurellaceae in vivo*, due to its adaptation to the mouse, the animal species most easily and frequently used in experimental studies.

The aim of the present investigation was to analyze if *[P*.*] pneumotropica* biotypes Jawetz and Heyl and *[A*.*] muris* produce a biofilm and, if so, to look if this phenomenon is prevalent among the field isolates. Moreover, we aimed to characterize the thickness and the chemical composition of the biofilm and to test whether the susceptibility to antibiotics in biofilms was different to that of the planktonic cells for these obligate inhabitants of the mouse mucosa. We demonstrate for the first time that both *[P*.*] pneumotropica* biotypes but not *[A*.*] muris* are able to form robust biofilms *in vitro*, which seems to consist predominantly of proteins and extracellular DNA (eDNA). Moreover, we reveal that this phenotype is widely spread among the field isolates and might interfere with the sensitivity to antibiotics.

## Materials and Methods

### Bacterial strains and culture conditions

A total of 60 mouse *Pasteurellaceae* strains (Table 1), consisting of 17 isolates of *[P*.*] pneumotropica* biotype Jawetz (including the type strain Frederiksen P421^T^ = CCUG12398^T^), 25 isolates of *[P*.*] pneumotropica* biotype Heyl (including the type strain ATCC12555^T^) and 18 *[A*.*] muris* isolates (including the type strain CCUG16938^T^) were used in this study. The clinical strains were isolated during the periodically health monitoring [30] of the mice from the Animal Research Facility of the Heinrich-Heine-University in Düsseldorf and identified by standard biochemical tests as well as by the 16S–23S rDNA internal spacer profile and PCR as described previously [5, 21, 31]. The isolates were propagated from -80°C stocks onto Columbia blood-agar plates (Biomerieux, Nuertingen, Germany) for approximately 30 h at 37°C under aerobic conditions. Isolated colonies were further inoculated in brain heart infusion (BHI) broth (Sigma-Aldrich, Steinheim, Germany), overnight (approx. 16–18 h) at 37°C. The overnight cultures were used as inoculum for the following experiments.

**Table 1:** Bacterial strains used in this study.

Organism	Strain	Reference
Pasteurellaceae (n = 60)		
*[Actinobacillus] muris* (n = 18)	CCUG16938^T^	Christensen *et al*., 2003 [9]
	1596/07, 450/10, 490/11, 622/11, 691/11, 693/11, 694/11, 696/11, 808/11, 1040/11, 1041/11, 1239/11, 1526/12, 1528/12, 1576/12, 1578/12, 143/13	Benga *et al*., 2012 [5], Benga *et al*., 2013 [20]
*[P*.*] pneumotropica* biotype Heyl (n = 25)	ATCC12555^T^	Christensen *et al*., 2003 [9]
	218/08, 314/08, 520/08, 1825/08, 450/10, 256/11, 490/11, 543/11, 566/11, 622/11, 666/11, 693/11, 696/11, 705/11, 1023/11, 1070/11, 1527/12, 1528/12, 1552/12, 526/13, 716/13, 717/13, 1378/13, 1379/13	Benga *et al*., 2012 [5], Benga *et al*., 2013 [30]
*[P]*. *pneumotropica* biotype Jawetz (n = 17)	P421^T^ = CCUG12398^T^	Christensen *et al*., 2003 [9]
	1596/07, 217/08, 607/10, 567/11, 665/11, 691/11, 695/11, 847/11, 1009/11, 1012/11, 1023/11, 394/12, 1526/12, 1528/12, 1550/12, 530/13	Benga *et al*., 2012 [5], Benga *et al*., 2013 [30]

### Biofilm assay in microtiter plates and glass tubes

To quantify biofilm formation in microtiter plates and glass tubes the protocols described previously by [32] and [33] respectively were adapted with some modifications. In brief, the bacterial strains were grown overnight in 10 ml BHI, then 20 μl culture was mixed with 2 ml fresh warm BHI in laboratory glass tubes 16 x 100 mm. Subsequently, 100 μl of the diluted culture was transferred in triplicate to 96-well plates (Rotilabo®-microtest plates, Carl-Roth, Karlsruhe, Germany). The 96-well plates and the glass tubes with the remaining culture were incubated aerobe, under static conditions for 24 h at 37°C, when the culture medium was removed and the recipients were rinsed once with tap water (pH 7.3). Subsequently, 100 μl or 2 ml of 1% crystal violet dye (Merck, Darmstadt, Germany) was added to each well and glass tube respectively for 15 min at room temperature. Following, the plates and the tubes were washed three times with water and the excess water was removed from the plates and glass tubes by tapping the inverted recipients on paper towel. The glass tubes were allowed to dry and then photographed, whereas the biofilm formed in the 96-well plates was quantified by recording the absorbance at 540 nm after addition of 100 μl of 70% ethanol for 15 min at room temperature to solubilize the crystal violet. Wells containing only BHI were used as controls and their OD_540_ values were subtracted from all the samples. Glass tubes containing sterile BHI were included as well as controls and compared with the inoculated tubes.

### Confocal laser scanning microscopy (CLSM)

For microscopic visualization of the biofilms, overnight BHI broth cultures of the type strains of *[P*.*] pneumotropica* biotypes Jawetz and Heyl and of *[A*.*] muris* were diluted 1:100 in fresh BHI broth. Subsequently, 7 ml of the dilution was placed in Ф 5.5 cm Petri dishes (Greiner, Solingen, Germany) containing glass cover slips for 24 h at 37°C under aerobe conditions, then the culture supernatant was replaced and the biofilms were gently rinsed with distilled water. Each cover slip was then treated with 4% formalin for 5 min at room temperature and the excess formalin was rinsed with water. The biofilms were then stained with 0.2% water solution of acridine orange (Sigma-Aldrich), which labels double-stranded nucleic acids in green and single-stranded nucleic acids in red, for 5 min as previously described [34]. The excess stain was gently rinsed off and the cover slips were mounted up-side down using fluoromont^TM^ aqueous mounting medium (Sigma-Aldrich). Confocal images were acquired with a Zeiss LSM710 Confocal Microscope (Carl Zeiss, Jena, Germany) using a Plan Apochromat 63X/1.4 objective. Stacks of horizontal plane images with a z-step of 1 μm were acquired. Orthogonal projections of the biofilms were constructed using the Zeiss confocal ZEN software.

### Biofilm inhibition and detachment assay

To find out whether proteins, DNA and polysaccharides were involved in the biofilm formation or are incorporated within the mature biofilms of *[P*.*] pneumotropica*, we performed biofilm inhibition and detachment assays respectively in the presence of (i) proteinase K (Sigma-Aldrich), (ii) DNase I (Sigma-Aldrich) and (iii) sodium *meta*-periodate (NaIO_4_) (Sigma-Aldrich) as major protein, DNA and β-1,6-linked polysaccharide inhibitors as previously described [35, 36] with some modifications. For the inhibition assays, overnight cultures in BHI were diluted 1:100 in BHI containing (i) 100, 50, 25, 12.5 or 6.25 μg/ml proteinase K, (ii) 100, 50, 25, 12.5 or 6.25 μg/ml DNase I, or (iii) 10, 5, 2.5, 1.25 or 0.625 mM sodium periodate and allowed to form biofilms for 24 h at 37°C whereupon the biofilm quantity was recorded by the crystal violet assay described above. Wells Wells containing BHI without any supplements were used as controls. For detachment experiments, the culture supernatant from the wells containing 24 h old biofilms was replaced for 2 h at 37°C by 100 μl (i) proteinase K, (ii) DNase I or (iii) sodium periodate (NaIO_4_) at the concentrations listed above. Proteinase K was solubilized in a buffer containing 20 mM Tris-HCl (pH 7.5) – 100 mM NaCl, whereas the DNase I and sodium periodate were solubilized in 150 mM NaCl – 1 mM CaCl_2_ and 50 mM sodium acetate respectively. Wells treated with the corresponding buffers without the active substances were used as controls. Finally, the wells were washed three times with tap water and the biofilm was quantified using the crystal violet assay.

### Antibiotic susceptibility of planktonic and biofilm bacteria

The susceptibility of biofilm versus planktonic bacteria to amoxicillin (Sigma-Aldrich) and enrofloxacin (Baytril, Bayer, Leverkusen, Germany) was assessed by recording the minimal inhibitory concentrations (MICs) and by a viability cfu plating assay as previously described [37, 38], using the type strains of the *[P*.*] pneumotropica* biotypes Jawetz and Heyl as well as an additional field isolate (394/12 and 1070/11) of each biotype originating from abscesses. Briefly, to determine the MICs for planktonic bacteria, overnight cultures were diluted to OD_540_ = 0.15 in BHI containing two-fold serial dilutions (200–0.19 μg/ml) of antibiotic and 200 μl of the suspensions were transferred in 96-well plates. For biofilms, the supernatants of 24 h old biofilms of the same strains were replaced by 200 μl BHI containing the same concentrations of antibiotic as for the planktonic cells. After incubation for 24 h at 37°C the growth was recorded by measurement of absorbance at 540 nm. The experiments were repeated at least three times. The MIC was considered the lowest concentration at which no statistically significant growth occurred after 24 h. For the viability cfu plating assays [37], the supernatants of 24 h old biofilms prepared in 96-well microtiter plates as described above were replaced with 200 μl of either fresh BHI broth or of BHI containing one of the antibiotics at a concentration of 100 μg/ml. After 3 h incubation at 37°C, the wells were washed three times with 250 μl sterile 0.9% NaCl. The biofilms were then removed by thoroughly scraping of the wells and suspended in 1 ml 0.9% NaCl. The viable cell number was determined by performing 10-fold serial dilution, plating and cfu enumeration after 24 h incubation at 37°C. To determine the susceptibility of planktonic bacteria to antibiotics, a fivefold dilution of overnight cultures in BHI followed by 3 h incubation at 37°C was performed. Subsequently, 2 ml of this culture were added to 10 ml BHI broth containing each antibiotic at a concentration of 100 μg/ml. A control dilution was obtained by diluting the suspension in BHI without antibiotics. Growth was allowed to occur over 3 h at 37°C when 1 ml culture was harvested and centrifuged for 8 min at 9,000 g. The pellet was then resuspended in 1 ml 0.9% NaCl and washed three times using the same procedure. Finally, the number of viable cells was determined by the same procedure used for the biofilm. These experiments were repeated at least three times, in duplicates.

### Statistical analysis

For the inhibition and dispersal assays as well as for the bacterial growth, variance analysis followed by two-tailed Student´s *t* test was used to determine significance in the differences between the treated versus control groups. Differences were considered significant when confidence levels >95% were achieved.

## Results

### *[P*.*] pneumotropica* biotypes Heyl and Jawetz but not *[A*.*] muris* isolates form robust biofilms

**Fig 1:**
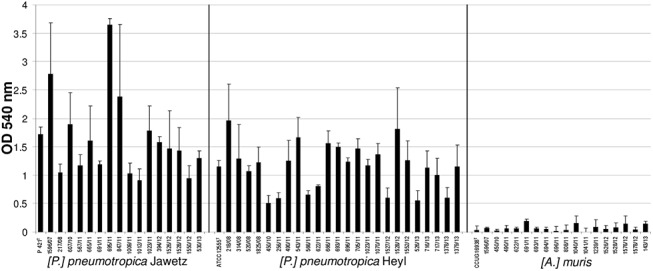
Biofilm formation in different rodent Pasteurellaceae strains. The biofilm formation was recorded after 24 h of growth by a standard crystal violet microtiter plate assay and measuring the absorbance at 540 nm (y-axis). Strains tested are shown on the x-axis and grouped by species. Bars represent the average absorbance + standard deviation from three independent experiments.

**Fig 2:**
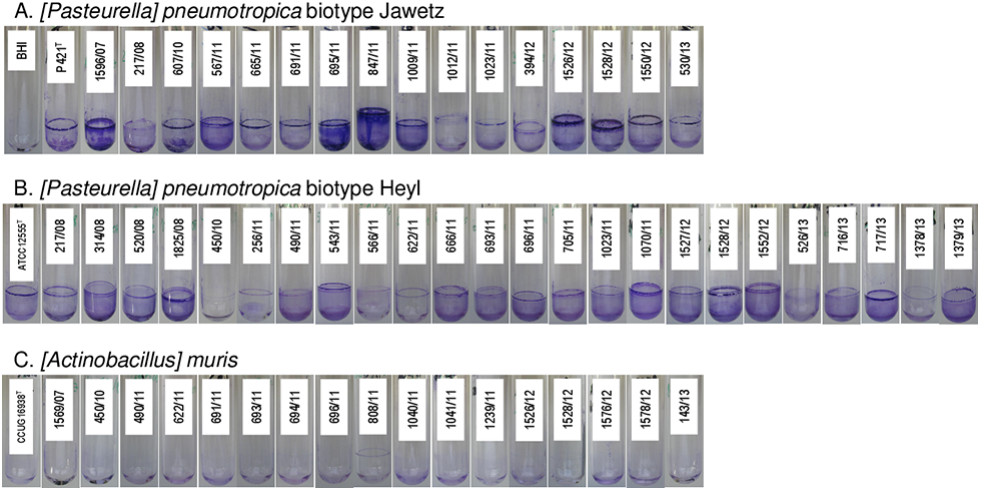
Biofilm formation by rodent Pasteurellaceae in glass tubes. Representative pictures of biofilm formation in glass tubes by *[P*.*] pneumotropica* biotype Jawetz (A.), *[P*.*] pneumotropica* biotype Heyl (B.) and *[A*.*] muris* (C.) after 24 h incubation at 37°C and staining with crystal violet. The strain number is pasted on the corresponding tube. The picture of a tube containing sterile BHI was included in panel A for comparison purposes.

**Fig 3:**
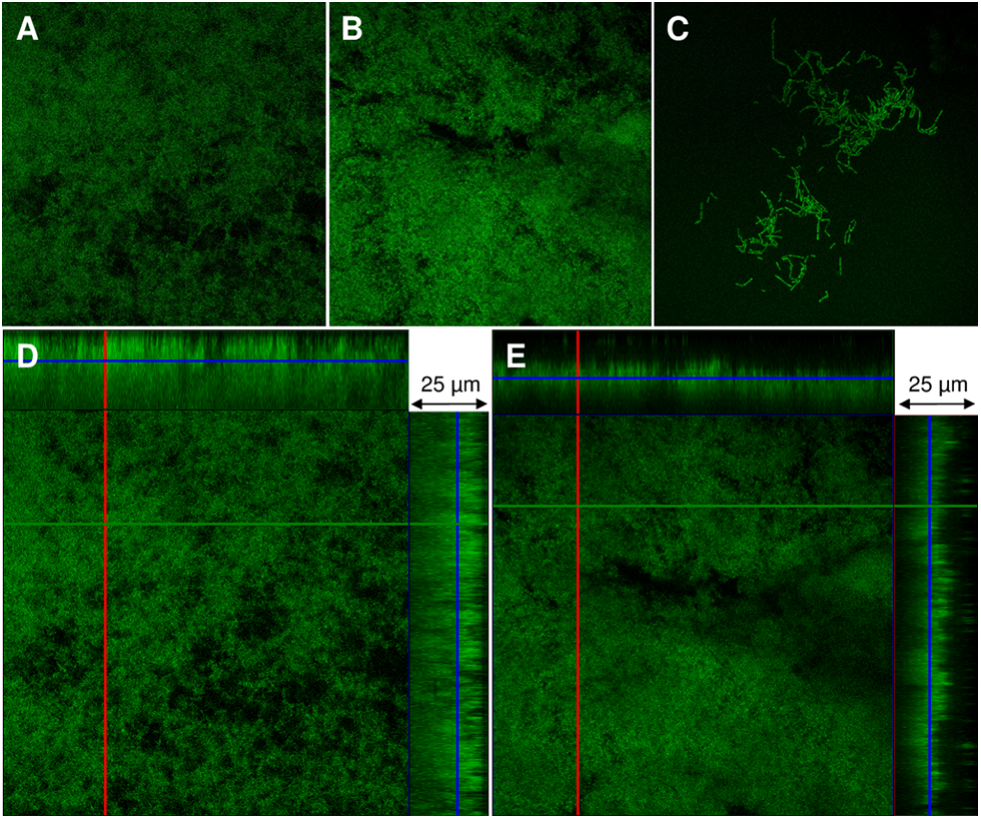
Confocal laser scanning microscopy analysis of rodent *Pasteurellaceae* biofilms. The type reference strains of the three rodent *Pasteurellaceae* species studied were allowed to produce biofilms for 24 h on glass coverslips and then examined by CLSM as described in Materials and Methods. The upper panel images are two-dimensional images of the biofilms formed by [P.] pneumotropica biotype Jawetz (A), *[P*.*] pneumotropica* biotype Heyl (B) and *[A*.*] muris* (C). The lower panels are orthogonal views of z-stacks of *[P*.*] pneumotropica* biotype Jawetz (D) and *[P*.*] pneumotropica* biotype Heyl biofilms (E) where the larger panel is a “bird´s eye” view of the biofilms whereas the right and the upper panels are side views of x- and y-axis sections respectively.

To date, it is not known whether the three rodent *Pasteurellaceae* species included in this study are able to produce biofilms. To address this, biofilm formation was quantified by a standard microtiter plate crystal violet assay as well as by a glass tube assay in a various collection of reference and field isolates of *[P*.*] pneumotropica* biotypes Jawetz and Heyl and *[A*.*] muris*. Fig 1 shows the amount of biofilm formed after 24 h in BHI as recorded photometrical using the crystal violet technique. There is an obvious difference in the biofilm formation of *[P*.*] pneumotropica* species belonging to both biotypes in comparison to the *[A*.*] muris* isolates which can be categorized as no biofilm producers. In sharp contrast to *[A*.*] muris*, all *[P*.*] pneumotropica* strains tested were capable to produce robust biofilms, even though one can differentiate among them strong and weak biofilm producers. The results obtained by the glass tubes biofilm formation assays correlated with the results obtained by the crystal violet microtiter plate assays, showing as well the ability of *[P*.*] pneumotropica* biotypes Jawetz and Heyl but not of *[A*.*] muris* to form biofilms (Fig 2). Confocal microscopy analyses using the type reference strains of *[P*.*] pneumotropica* biotypes Jawetz and Heyl and of *[A*.*] muris* showed that indeed only *[P*.*] pneumotropica* strains, but not *[A*.*] muris*, were able to produce consistent biofilms (Fig 3).

### Inhibition of biofilm formation by proteinase K, DNase I and sodium periodate

Proteins, DNA and polysaccharides may play an important role in the extracellular matrix of bacterial biofilms. To address the functional role of these components in the *[P*.*] pneumotropica* biofilm formation, we studied the ability of proteinase K, DNase I and sodium periodate to inhibit biofilm development. For this, the components listed above were added into the growth media at the time of bacterial inoculation. Subsequently the cultures were allowed to develop biofilms for 24 h at 37°C.

**Fig 4:**
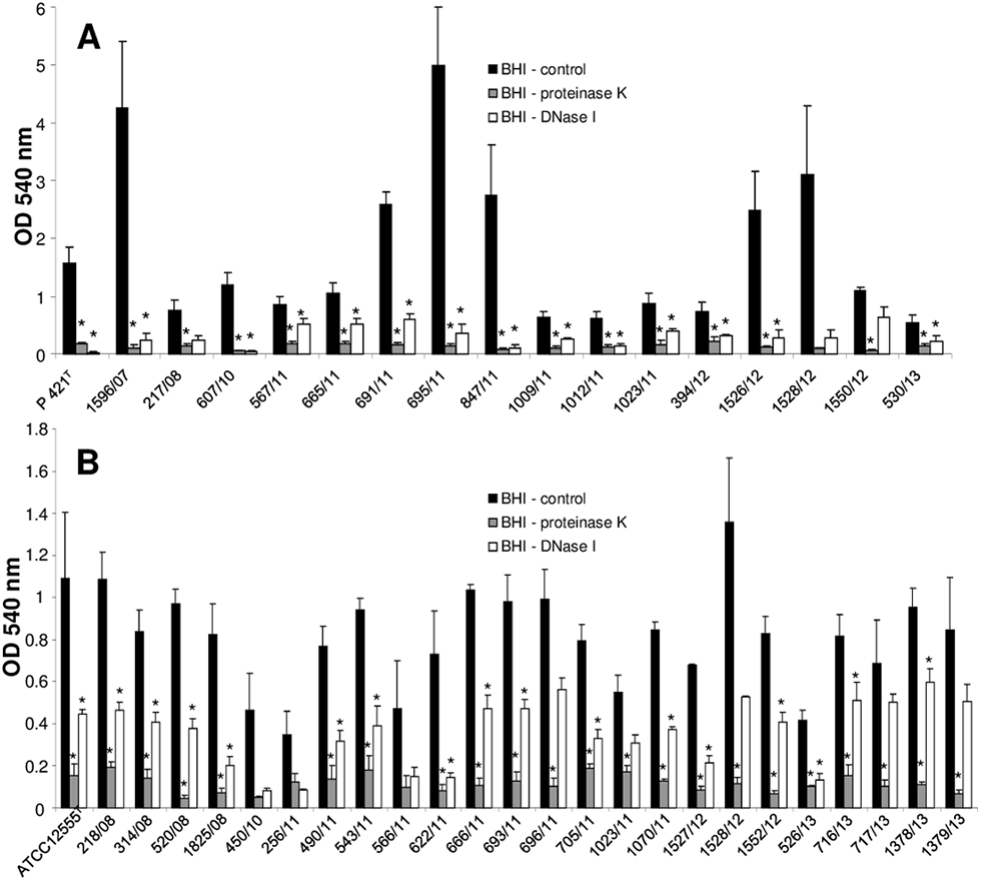
Inhibition of biofilm formation by proteinase K and DNase I in *[P*.*] pneumotropica* biotypes Jawetz (A) and Heyl (B). The bacterial strains indicated along the x-axis were grown statically for 24 h in BHI broth alone (black bars) or in BHI broth supplemented with 100 μg/ml proteinase K (grey bars) or 50 μg/ml DNase I (white bars). The biofilm formation was then quantified photometrical at 540 nm after staining with crystal violet. Bars represent mean values + standard deviation of at least three independent experiments. Asterisks (*) designate a p-value less than 0.05 between the treated group and the corresponding control.

Cultures of the type reference strains of *[P*.*] pneumotropica* biotypes Jawetz und Heyl in BHI containing a range of proteinase K concentrations from 100 to 6.25 μg/ml did not affect the growth of bacteria (S1 Fig). Interestingly, a proteinase K dose dependent inhibition in the formation of biofilms was observed for both type strains of *[P*.*] pneumotropica* used (S1 Fig). To test whether the proteinase K inhibition in the biofilm formation is spread among *[P*.*] pneumotropica* field isolates or was a specific characteristic of the reference strains, we tested the effect of 100 μg/ml proteinase K on all *[P*.*] pneumotropica* isolates included in this study. All but one of the 17 *[P*.*] pneumotropica* biotype Jawetz isolates were significantly inhibited in their ability to form biofilm by proteinase K (Fig 4A). Twenty-two of the 25 *[P*.*] pneumotropica* biotype Heyl isolates displayed as well a statistically significant reduction in the biofilm formation in the presence of proteinase K (Fig 4B). The p-values for the isolates 1528/12, 450/10, 256/11 and 566/11 which did not show statistically significant differences were 0.06, 0.08, 0.14 and 0.09.

Introduction of serial two-fold dilutions of DNase I (from 100 to 6.25 μg/ml) into the growing medium did not affect the growth of *[P*.*] pneumotropica* isolates. Similar to the proteinase K, a DNase I dose dependent inhibition in the biofilm formation was observed for the type reference strains (S2 Fig). In addition, the biofilm formation was significantly impaired by the presence of 50 μg/ml DNase I in the growth medium, by all but three of the *[P*.*] pneumotropica* biotype Jawetz strains (Fig 4A) and in all but eight *[P*.*] pneumotropica* biotype Heyl strains (Fig 4B) tested. Although no statistical significant differences were observed for some strains, probably due to the high standard deviation of the control group, a clear inhibition tendency in biofilm formation was observed (Fig 4) with p-values between 0.05 and 0.1 for all but one of the eleven strains.

To evaluate the role of β-1,6-linked polysaccharides in the biofilm formation, the two *[P*.*] pneumotropica* type reference strains were allowed to form biofilms in BHI containing two fold serial dilutions of 10 to 0.625 mM sodium periodate. Even the lowest concentration of sodium periodate (0.625 mM) inhibited the growth of *[P*.*] pneumotropica* biotype Jawetz, thus the reduction in the biofilm formation by 2.5 mM sodium periodate could be attributed to the impaired growth of bacteria (S3 Fig). A similar reduction of the biofilm formation at reduced bacterial growth displayed the type strain of *[P*.*] pneumotropica* biotype Heyl (S3 Fig). Due to the growth inhibition of the type strains by sodium periodate, no further tests using additional isolates were performed in these experimental settings.

### Dispersal of biofilms by proteinase K, DNase I and sodium periodate

To characterize in more detail the role of proteins, DNA and polysaccharides in the structure of mature biofilms, we tested the capability of proteinase K, DNase I and sodium periodate to disperse pre-formed mature biofilms. For this, the same components used for the inhibition of biofilm formation were applied for 2 h on 24 h old biofilms and their ability to disperse biofilm in comparison to non treated controls was recorded.

**Fig 5:**
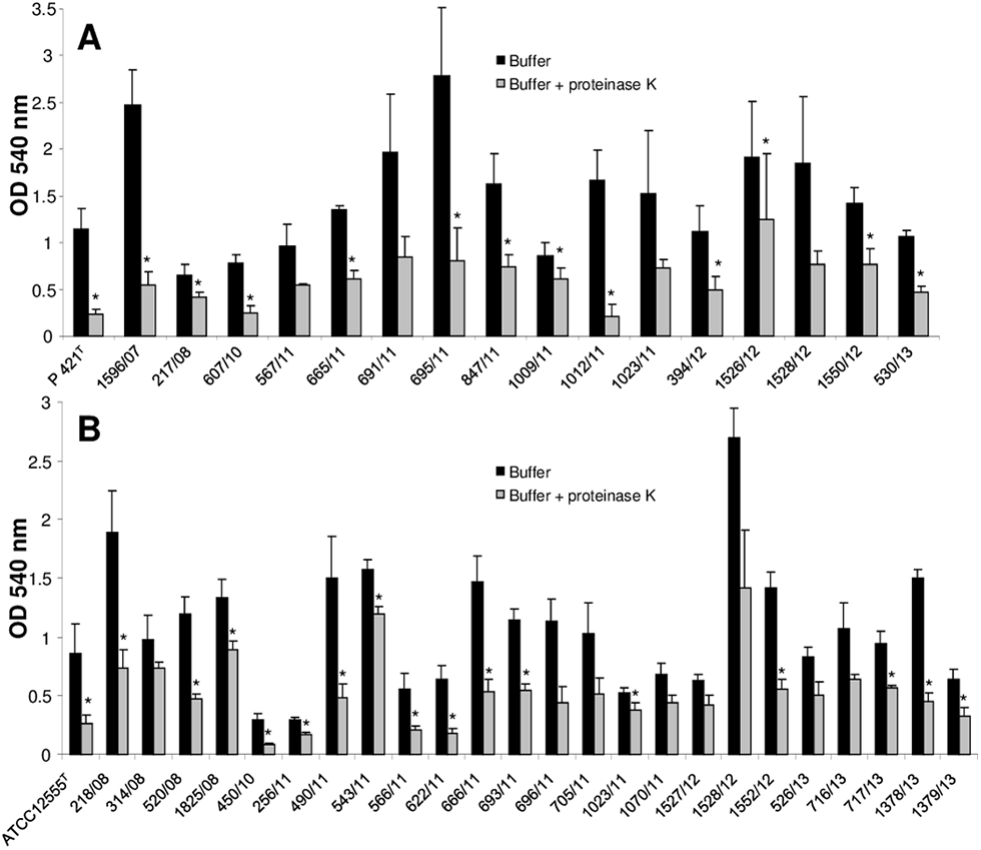
Dispersal of mature biofilms of *[P*.*] pneumotropica* biotypes Jawetz (A) and Heyl (B) by proteinase K. The supernatants of 24 h old biofilms of the strains indicated along the x-axis were replaced for 2 h by buffer alone (black bars) or buffer containing 100 μg/ml proteinase K (grey bars). Biofilm quantity was then recorded using a standard crystal violet assay by measuring the absorbance at 540 nm. Bars represent mean values + standard deviation of at least three independent experiments. Asterisks (*) designate a p-value less than 0.05 between the treated group and the corresponding control.

Concentrations of 100 μg/ml of proteinase K were necessary to achieve a statistically significant disruption of the biofilm produced by both *[P*.*] pneumotropica* type strains in comparison with the non-treated controls (S1 Fig). Incubation of the mature biofilms from the field isolates of *[P*.*] pneumotropica* with 100 μg/ml proteinase K caused significant detachment in nearly all strains tested (Fig 5). By four strains of *[P*.*] pneumotropica* biotype Jawetz and eight strains of *[P*.*] pneumotropica* biotype Heyl no statistically significant (p—values between 0.06 and 0.14 and one value of 0.22) proteinase K dispersal of the biofilms was achieved, although visual differences could be recognized.

**Fig 6:**
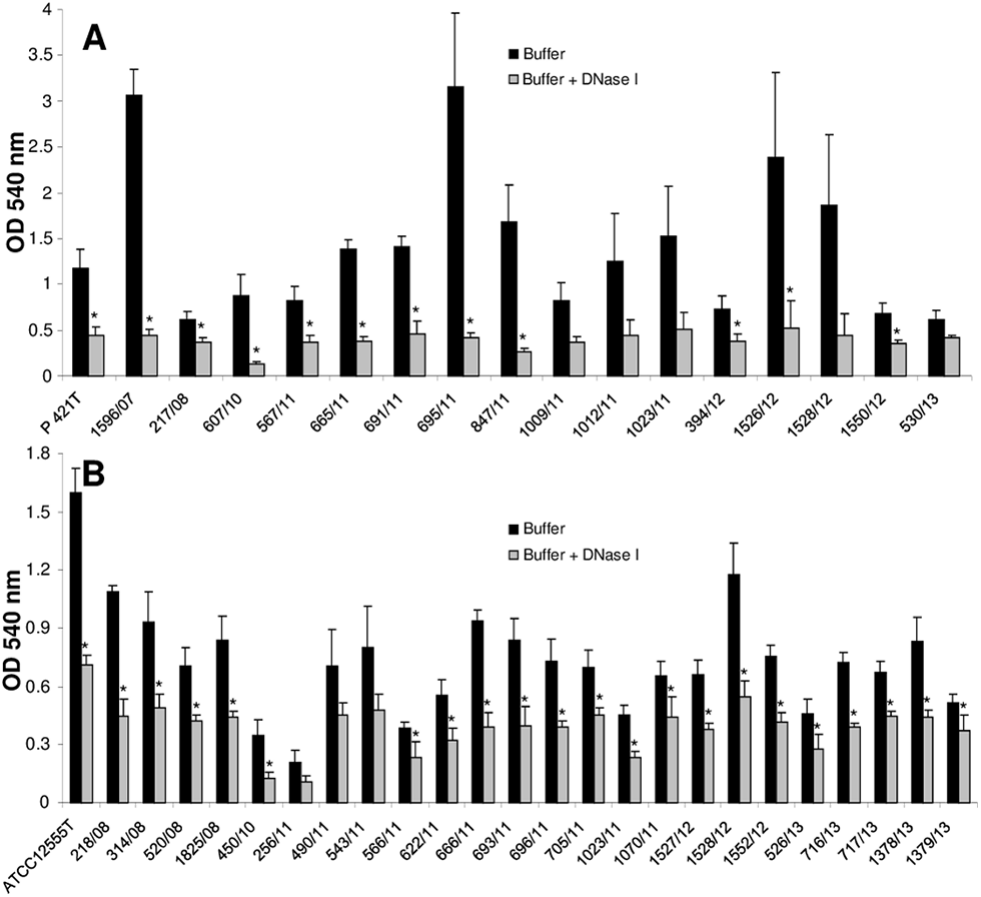
Dispersal of mature biofilms of *[P*.*] pneumotropica* biotypes Jawetz (A) and Heyl (B) by DNase I. The supernatants of 24 h old biofilms of the strains indicated along the x-axis were replaced for 2 h by buffer alone (black bars) or buffer containing 50 μg/ml DNase I (grey bars). Biofilm quantity was then recorded using a standard crystal violet assay by measuring the absorbance at 540 nm. Bars represent mean values + standard deviation of at least three independent experiments. Asterisks (*) designate a p-value less than 0.05 between the treated group and the corresponding control.

Treatment of the pre-formed biofilms with DNase I had a similar dispersal effect compared with proteinase K. S2 Fig shows that even 12.5 μg/ml DNase I was enough to cause a significant dispersal of the biofilm in the two type strains of *[P*.*] pneumotropica* biotypes Jawetz and Heyl. Notably, all but eight *[P*.*] pneumotropica* isolates tested were statistically sensitive to dispersal by 50 μg/ml of DNase I (Fig 6). For the remaining eight strains p-values between 0.06 and 0.09 were recorded.

**Fig 7:**
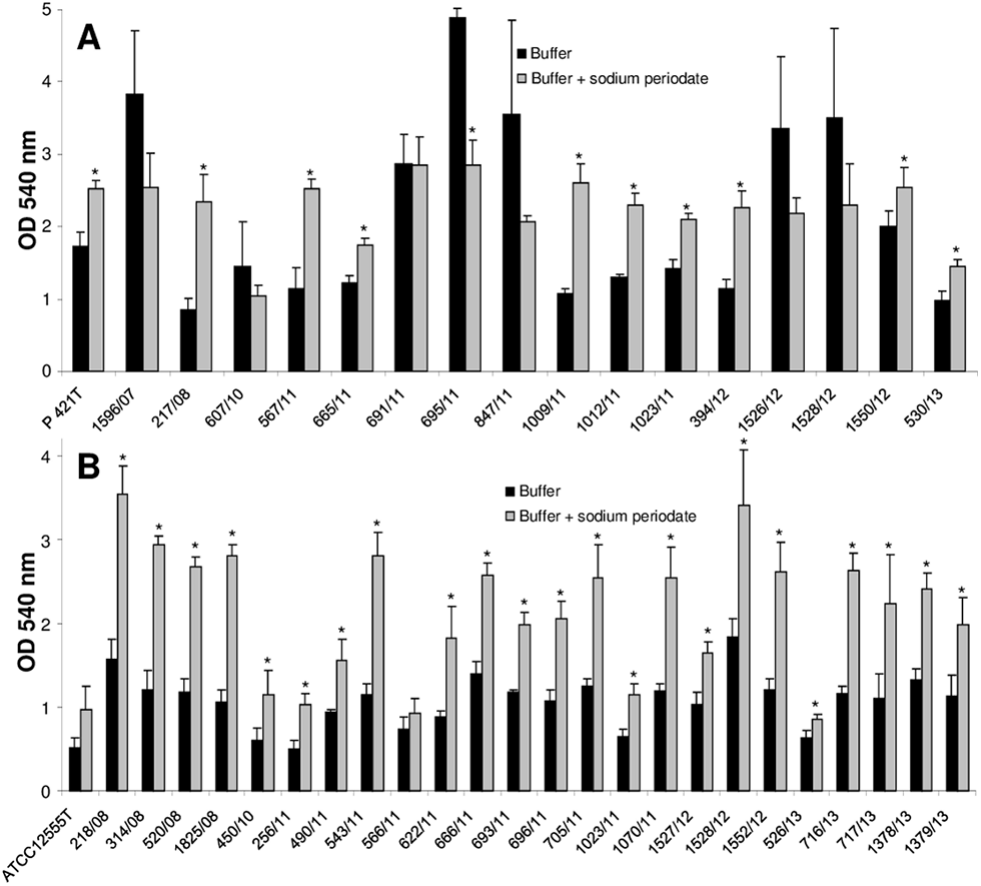
Dispersal of mature biofilms of *[P*.*] pneumotropica* biotypes Jawetz (A) and Heyl (B) by sodium periodate. The supernatants of 24 h old biofilms of the strains indicated along the x-axis were replaced for 2 h by buffer alone (black bars) or buffer containing 10 mM sodium periodate (grey bars). Biofilm quantity was then measured by a standard crystal violet assay and measuring the absorbance at 540 nm. Bars represent mean values + standard deviation of at least three independent experiments. Asterisks (*) designate a p-value <0.05 between the treated group and the corresponding control.

Treatment of the pre-formed biofilms of the reference strains with sodium periodate resulted surprisingly in an increase in the biofilm mass at already 5 mM in the case of *[P*.*] pneumotropica* biotype Jawetz and at 10 mM in the case of *[P*.*] pneumotropica* biotype Heyl (S3 Fig). However, treatment of the *[P*.*] pneumotropica* biotype Jawetz isolates with 10 mM sodium periodate had a varying effect on biofilm dispersal. Six of the 17 strains tested did not show any modification in the biofilm amount after treatment with sodium periodate, whereas a significantly reduced amount of biofilm could be measured in strain 695/11. The remaining ten isolates displayed an increase in the biofilm quantity after treatments with sodium periodate (Fig 7A). The phenomenon of increase in the biofilm amount, after treatment with 10 mM sodium periodate was also observed for all but two of the *[P*.*] pneumotropica* biotype Heyl strains tested (Fig 7B).

### Antibiotic susceptibility of planktonic and biofilm bacteria

**Fig 8:**
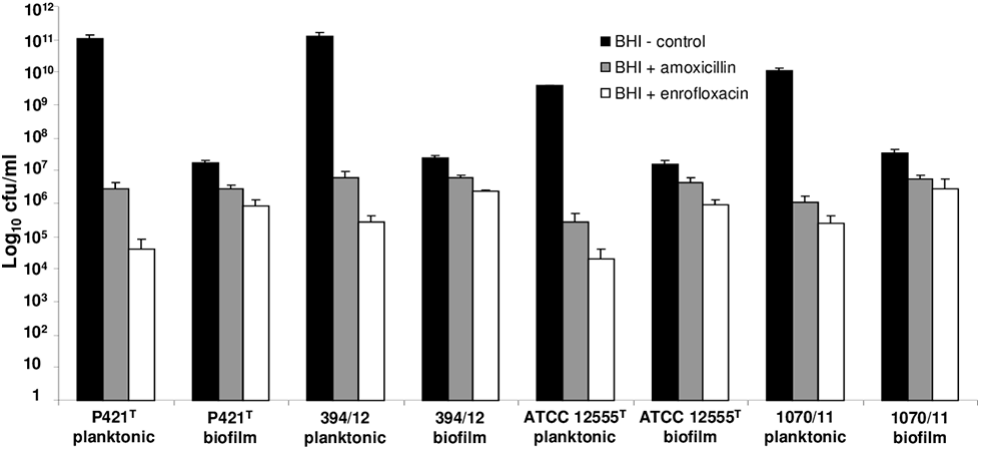
Reduction of cellular viability of planktonic and biofilm cells of *[P*.*] pneumotropica* after 3 hours exposure to antibiotics. The y-axis indicates the log_10_ cfu/ml recovered from the controls without antibiotics (black bars) in comparison to bacteria treated with amoxicillin (grey bars) or enrofloxacin (white bars) in planktonic or biofilm status. Bars represent mean values + standard deviation of three independent experiments.

The MICs of amoxicillin to all *[P*.*] pneumotropica* strains used were higher for the bacteria grown in biofilms than for planktonic cells (Table 2). The MICs of enrofloxacin was similar for both biofilm and planktonic cells in three of the strains used (ATCC12555^T^, CCUG12398^T^, 394/12), whereas for the strain 1070/11 it was higher for the bacteria grown in biofilm (Table 2). Fig 8 presents the reduction in viability of selected *[P*.*] pneumotropica* strains after exposure of planktonic cells or biofilms to amoxicillin and enrofloxacin in comparison to non treated controls. The antibiotics treatment reduced significantly the number of viable cells in both planktonic and biofilm cells in comparison to the non treated controls. However, the viable cells from the antibiotics treated samples were outnumbered by the control cells by a factor of about 10:1 in the case of biofilms and by a factor of about 10^5^:1 in the case of planktonic cells, showing thus a more severe reduction in the cellular viability for the planktonic in comparison to biofilm bacteria.

**Table 2:** Minimal inhibitory concentrations (MICs) of amoxicillin and enrofloxacin for *[P*.*] pneumotropica* in planktonic and biofilm status.

Strain	Growth	Antibiotic	
		Amoxicillin (μg/ml)	Enrofloxacin (μg/ml)
ATCC12555^T^	Biofilm	12.5	0.78
	Planktonic	<0.19	0.78
1070/11	Biofilm	100	12.5
	Planktonic	<0.19	<0.19
CCUG12398^T^	Biofilm	100	<0.19
	Planktonic	12.5	<0.19
394/12	Biofilm	25	<0.19
	Planktonic	<0.19	<0.19

## Discussion

Biofilm formation is spread among many bacterial pathogens and plays an important role in the pathogenesis of disease and protection from therapy [4]. To date it is known that many facultative pathogenic members of the *Pasteurellaceae* family such as *P*. *multocida* [39], *A*. *pleuropneumoniae* [33], *A*. *actinomycetemcomitans* [40], *H*. *influenzae* [41], *H*. *somni* [1] and *H*. *parasuis* [42] are able to form biofilms. In the present investigation we analyzed the capacity of *[P*.*] pneumotropica* biotypes Jawetz and Heyl, which can be regarded as two different species [9] and of *[A*.*] muris* to form biofilms *in vitro*. One can depict at first glance from the Figs 1 and 2 that the two *[P*.*] pneumotropica* biotypes species strains are able to form robust biofilms, in contrast to *[A*.*] muris* isolates which are no biofilm producers under the same experimental conditions. In correlation with the photometrical results, the CLSM imagines definitively show that only *[P*.*] pneumotropica* strains but not *[A*.*] muris* are able to produce biofilms with a thickness of about 20 μm under the conditions used in this study (Fig 3). Although all three species used in this study belong to the normal murine respiratory tract microbiota as shown recently [43], only *[P*.*] pneumotropica* biotypes Jawetz and Heyl possess a known pathogenic potential [13, 16, 44], whereas *[A*.*] muris* is considered to be non-pathogenic [21], suggesting that biofilm production might represent a virulence attribute for these bacteria.

To further characterize the mechanisms involved in the biofilm formation and their chemical composition, we tested whether substances targeting major components of the biofilm matrix like proteins, eDNA or polysaccharides could inhibit the biofilm formation or affect established biofilms. Enzymes like proteinase K, DNase I or compounds targeting β-1,6-linked polysaccharides such as sodium periodate have different kinds of actions on particular types of biofilms [35, 45].

Our study demonstrates that proteins are essential in biofilm formation in both *[P*.*] pneumotropica* biotypes, since the proteinase K at a concentration that does not affect cell growth, clearly inhibited the biofilm formation (Fig 4). Dispersal of the pre-formed biofilms by the proteinase K treatment demonstrates, using a different approach, that proteins are essential components of the *[P*.*] pneumotropica* biofilms (Fig 5). Due to their localization on the surface of bacteria, it is plausible that proteins play an important role in colonization and biofilm formation. Families of surface associated proteins involved in adherence and biofilm formation such as the biofilm-associated proteins (Bap), containing a core domain of tandem repeats that confer bacteria the ability to form a biofilm, seem to be present in many species of Gram-positive and Gram-negative bacteria [46]. In correlation with our findings, proteins have been shown to play a central role in the biofilms of other species belonging to the family of *Pasteurellaceae* such as *Haemophilus parasuis* [47] or in important human and animal pathogens such as MRSA [48].

DNase I at concentrations that do not affect bacterial growth, inhibited biofilm formation and dispersed preformed biofilms of *[P*.*] pneumotropica* (Figs 4 and 6), suggesting that eDNA similarly to proteins plays a decisive role in the biofilm construction. This finding is in agreement with other studies which also showed the importance of eDNA in biofilm formation for other related or not related bacteria [45, 47–49]. Autolysis is the common mechanism by which eDNA is released. eDNA has a crucial role in stabilizing biofilm structures and represents an important mechanism for horizontal gene transfer in bacteria. Furthermore, it is recognized by the innate immune system via the TLR family receptors [50].

It seemed that sodium periodate did not disturb the biofilm formation of the type reference strains of *[P*.*] pneumotropica* biotypes when added to the growth medium. However, the growth of the type strains of *[P*.*] pneumotropica* was inhibited by this substance (S3 Fig), thus making an objective evaluation of the role of β-1,6-linked polysaccharides in the biofilm formation in this experimental setting difficult. Consequently, no further inhibition experiments with additional bacterial isolates were performed. Interestingly, treatments of preformed biofilms of *[P*.*] pneumotropica* with sodium periodate produced varying effects. On one hand, this resulted in an increased biofilm production in all *[P*.*] pneumotropica* biotype Heyl and 10 out of 17 *[P*.*] pneumotropica* biotype Jawetz isolates (Fig 7). On the other hand, sodium periodate had no effect or even dispersed the biofilm in some strong biofilm producer isolates of *[P*.*] pneumotropica* biotype Jawetz. Capsular polysaccharides seem generally to interfere with the contact of bacteria to surfaces, by masking adhesive structures of other biochemical structure. For example, the capsule impairs the adherence of *Streptococcus suis* to epithelial cells [51]. Similarly, capsule reduction correlated with increased biofilm formation by some *Streptococcus agalactiae* strains [49]. Moreover, the capsule of *A*. *pleuropneumoniae* possess antibiofilm activity against both Gram-positive and Gram-negative bacteria [52]. *[P*.*] pneumotropica* possess a slight extracellular fibrous material associated with the cell wall when stained with ruthenium red [53]. One can speculate that the treatment of *[P*.*] pneumotropica* biofilms with sodium periodate and thus removal of the capsular polysaccharides lead to a better surface exposure of proteins important in biofilm formation and thus to an increased biofilm stability, more resistant to the washing steps which preceded the quantification of the biofilm amount. In contrast, it seems that for the strong biofilm producers of the biotype Jawetz, the polymers of β-1,6-linked polysaccharides are integrated in the biofilms, reminding on structures similar to poly-β-1,6-*N*-acetyl-D-glucosamine (PGA) of *E*. *coli* [54], which are also present in *[P*.*] pneumotropica* related species such as *A*. *pleuropneumoniae* [55], *A*. *actinomycetemcomitans* [56] and *Haemophilus parasuis* [47] or in Gram-positive bacteria such as Staphylococci [57]. The variable behaviour of *[P*.*] pneumotropica* biotype Jawetz to sodium periodate suggests that different serotypes might be present within this species as known for other related *Pasteurellaceae* such as *A*. *pleuropneumoniae*. However, further investigations are necessary to confirm these suppositions.

Bacterial biofilms confer an increased resistance of bacteria to antibiotics and represent thus one of the reasons why treatment with antibiotics fails [3]. To study whether *[P*.*] pneumotropica* biofilms are able to interfere with the sensitivity to antibiotics we compared the MICs and the viability of planktonic and biofilm cells in the presence of a cell wall synthesis inhibitor (amoxicillin) and of a DNA gyrase inhibitor (enrofloxacin) antibiotic. Several mechanisms have been assumed to contribute to the biofilm resistance phenotype to antibiotics [37]. Our results demonstrate that the MICs of amoxicillin were higher for bacteria in biofilm than for planktonic cells. Similarly, the MIC of enrofloxacin was higher for the biofilm as for the planktonic cells in strain 1070/11. However, for the three other strains tested the MICs to enrofloxacin did not differ in biofilm and planktonic status (Table 2). Attempts to eliminate *[P*.*] pneumotropica* from mice colonies by treatment with enrofoxacin proved to be successful as demonstrated recently [58], showing the capacity of this antibiotic to neutralize *[P*.*] pneumotropica* cells. Unfortunately, the growth inhibition methods do not give information on the viability of the bacteria. In order to clarify whether the biofilm status confer as well a survival advantage in comparison to planktonic state, we used a cfu viability test. Using this assay, we demonstrate that *[P*.*] pneumotropica* cells enclosed in the biofilms were less sensitive than the planktonic cells to both antibiotics tested (Fig 8), which is in agreement with other studies [37, 38, 59]. Whether some of the known biofilm antibiotics resistance mechanisms contributed as well to this phenotype in the case of *[P*.*] pneumotropica* was not addressed here.

In conclusion, this is the first report showing that *[P*.*] pneumotropica* biotypes Jawetz and Heyl are strong biofilm producers and that this phenotype is uniform distributed among the field isolates. Moreover, we demonstrate that these biofilms, which are composed mainly of proteins and eDNA, displayed an increased resistance to antibiotics *in vitro* in comparison to planktonic bacteria. It is additionally hypothesized that surface polysaccharides might play a biofilm inhibitory role in *[P*.*] pneumotropica* biotype Heyl and in a majority of *[P*.*] pneumotropica* biotype Jawetz strains, whereas they might be integrated in the biofilm structure by some other Jawetz isolates. Notably, this study opens several questions to be addressed in the future. For example, further efforts are necessary to identify the proteins and other molecules that are decisive in the biofilm formation. It would be of interest as well to study whether the biofilm formation *in vitro* implies a similar phenotype on host cells as shown with *A*. *pleuropneumoniae* [60] and display an *in vivo* correspondent into the upper respiratory tract and the genital mucosa of mice. These would represent excellent tools to understand the biofilm mechanisms *in vivo* for a species specific pathogen such as *[P*.*] pneumotropica*, which could represent even a prototype for related microorganisms whose host specificities do not allow their study *in vivo* as easy as in the mouse model.

## Supporting Information

S1 Fig The effect of different concentrations of proteinase K on growth and biofilm formation by the type strains of *[P*.*] pneumotropica* biotypes Jawetz (A) and Heyl (B) and on the dispersal of mature biofilms of the same strains of biotypes Jawetz (C) and Heyl (D). For A and B, the strains P421^T^ and ATCC12555^T^ were allowed to grow and form biofilms in the presence of different concentrations (x-axis) of proteinase K. After 24 h the bacterial growth (grey bars) and subsequently the biofilm amount (black bars) of the same wells were quantified photometrical. To determine the biofilm dispersal capacity of proteinase K (C, D), bacterial growth (grey bars) was recorded on 24 h old biofilms. Subsequently, the wells were treated for 2 h with different concentrations of proteinase K (x-axis), washed and the biofilm amount (black bars) was recorded photometrical (540 nm) by a standard crystal violet assay. Average plus standard deviation of at least three independent experiments are shown. Asterisks (*) assign a p-value <0.05 between the treated groups and the non-treated control.
(TIFF)

S2 Fig The effect of DNase I on growth and biofilm formation (A, B) and on dispersal of pre-formed biofilms (C, D) of the type strains of *[P*.*] pneumotropica* biotypes Jawetz (A, C) and Heyl (B, D). The strains P421^T^ and ATCC12555^T^ were allowed to grow and form biofilms in the presence of different concentrations (x-axis) of DNase I (A, B). Bacterial growth (grey bars) and the biofilm amount (black bars) were recorded photometrical by 540 nm after 24 h. For C and D, bacterial growth (grey bars) was recorded on 24 h old biofilms. Subsequently, the wells were treated for 2 h with different concentrations of DNase I (x-axis), washed and the biofilm amount (black bars) was recorded photometrical (540 nm) by a standard crystal violet assay. Average plus standard deviation of at least three independent experiments are shown. Asterisks (*) designate a p-value <0.05 between the treated groups and the non-treated control.
(TIFF)

S3 Fig The effect of sodium periodate on growth and biofilm formation (A, B) and on dispersal of pre-formed biofilms (C, D) of the type strains of *[P*.*] pneumotropica* biotypes Jawetz (A, C) and Heyl (B, D). The strains P421^T^ and ATCC12555^T^ were allowed to grow and form biofilms in the presence of different concentrations (x-axis) of sodium periodate (A, B). Bacterial growth (grey bars) and the biofilm amount (black bars) were assessed 24 h later by measuring the absorbance at 540 nm. To evaluate the dispersal effect on pre-formed biofilms (C, D), the growth of 24 h old biofilms was recorded photometrical (grey bars). Subsequently, the biofilms were treated for 2 h with different concentrations of sodium periodate (x-axis), washed and the biofilm amount (black bars) was recorded photometrical (540 nm) by a standard crystal violet assay. Average plus standard deviation of at least three independent experiments are shown. Asterisks (*) assign a p-value <0.05 between the treated groups and the non-treated control.
(TIFF)
