# Supplementary figures and images for: Characterization of Biofilm Formation in [Pasteurella] pneumotropica and [Actinobacillus] muris Isolates of Mouse Origin
Source: PLoS One. 2015 Oct 2;10(10):e0138778. doi: 10.1371/journal.pone.0138778 (PMC4592018; doi:10.1371/journal.pone.0138778)

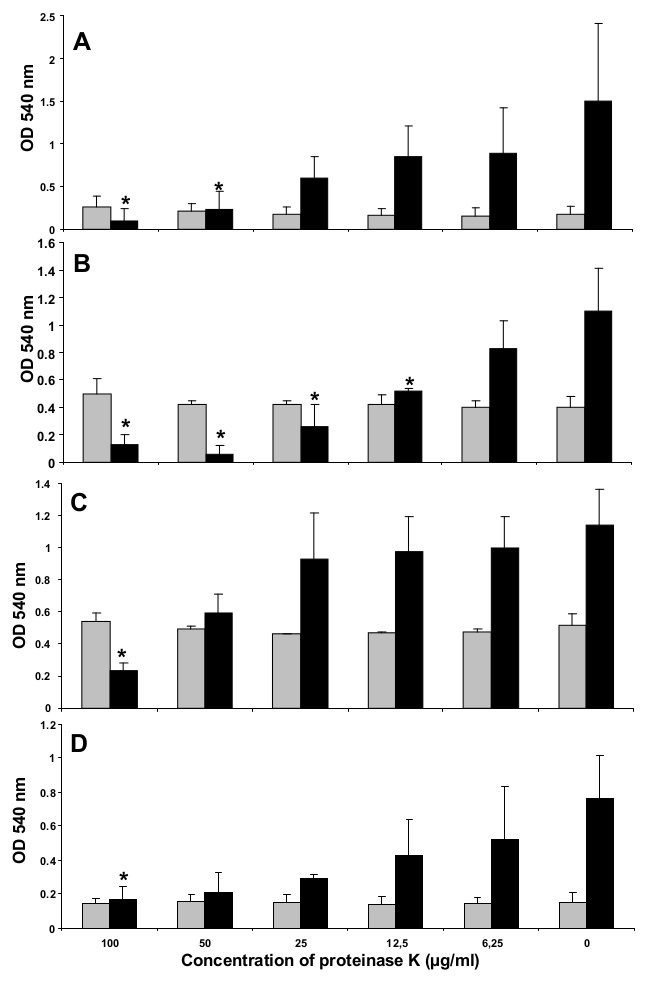

Supplement: S1 Fig — For A and B, the strains P421T and ATCC12555T were allowed to grow and form biofilms in the presence of different concentrations (x-axis) of proteinase K. After 24 h the bacterial growth (grey bars) and subsequently the biofilm amount (black bars) of the same wells were quantified photometrical. To determine the biofilm dispersal capacity of proteinase K (C, D), bacterial growth (grey bars) was recorded on 24 h old biofilms. Subsequently, the wells were treated for 2 h with different concentrations of proteinase K (x-axis), washed and the biofilm amount (black bars) was recorded photometrical (540 nm) by a standard crystal violet assay. Average plus standard deviation of at least three independent experiments are shown. Asterisks (*) assign a p-value <0.05 between the treated groups and the non-treated control. (TIFF) [file pone.0138778.s001.tiff]

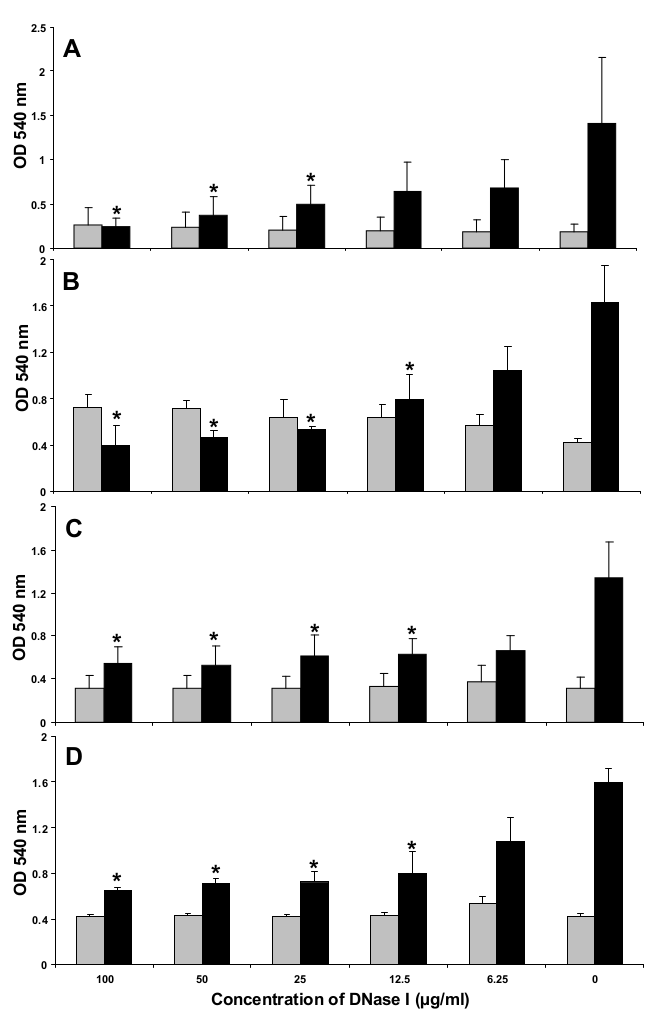

Supplement: S2 Fig — The strains P421T and ATCC12555T were allowed to grow and form biofilms in the presence of different concentrations (x-axis) of DNase I (A, B). Bacterial growth (grey bars) and the biofilm amount (black bars) were recorded photometrical by 540 nm after 24 h. For C and D, bacterial growth (grey bars) was recorded on 24 h old biofilms. Subsequently, the wells were treated for 2 h with different concentrations of DNase I (x-axis), washed and the biofilm amount (black bars) was recorded photometrical (540 nm) by a standard crystal violet assay. Average plus standard deviation of at least three independent experiments are shown. Asterisks (*) designate a p-value <0.05 between the treated groups and the non-treated control. (TIFF) [file pone.0138778.s002.tiff]

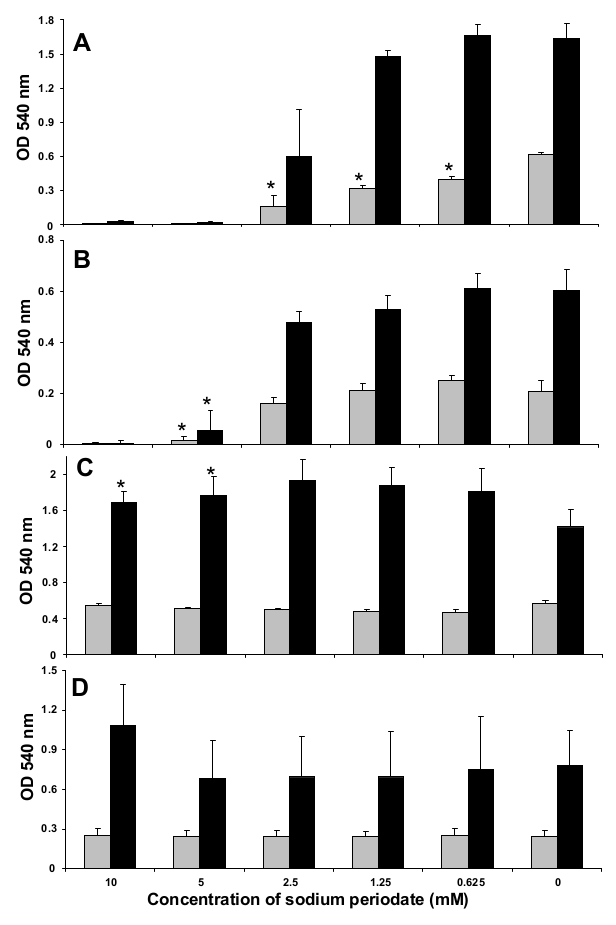

Supplement: S3 Fig — The strains P421T and ATCC12555T were allowed to grow and form biofilms in the presence of different concentrations (x-axis) of sodium periodate (A, B). Bacterial growth (grey bars) and the biofilm amount (black bars) were assessed 24 h later by measuring the absorbance at 540 nm. To evaluate the dispersal effect on pre-formed biofilms (C, D), the growth of 24 h old biofilms was recorded photometrical (grey bars). Subsequently, the biofilms were treated for 2 h with different concentrations of sodium periodate (x-axis), washed and the biofilm amount (black bars) was recorded photometrical (540 nm) by a standard crystal violet assay. Average plus standard deviation of at least three independent experiments are shown. Asterisks (*) assign a p-value <0.05 between the treated groups and the non-treated control. (TIFF) [file pone.0138778.s003.tiff]
